
JOURNAL INFORMATION
==============================
NLM Title Abbreviation: ScientificWorldJournal
Journal ID: ScientificWorldJournal
NLM Journal ID: 101131163
Journal ID: TSWJ

The Scientific World Journal

ISSN: 2356-6140
EISSN: 1537-744X
Publisher: Wiley

ARTICLE INFORMATION
==============================
PMCID: PMC6083844
PMID: 30147485
DOI: 10.1100/tsw.2001.23.144
Article ID: 247823
Article version: 1
Subjects: Short Report

Bcl-2 Suppresses Oxidative Phosphorylation Defects Caused by Mitochondrial DNA Mutations

Manfredi Giovanni 1 *
Kwong Jennifer Q. 1
Oca-Cossio Jose A. 2
Aurelio Marilena D. 1
Gajewsky Carl D. 1
Beal Flint M. 1
Moraes Carlos T. 2
Department of Neurology and Neuroscience, Weill Medical College of Cornell University, New York, NY 525 E 68th Street, A-505 New York, NY 10021, USA
2 Department of Neurology, Miami School of Medicine, University of Miami, Miami, FL, USA
*Giovanni Manfredi: gim2004@mail.med.cornell.edu
Publication date: 2001
Electronic publication date: 2001 Jul 17
Volume: 1
First page: 39
Last page: 39
Copyright: Copyright © 2001 Giovanni Manfredi et al.
Copyright year: 2001
License: This is an open access article distributed under the Creative Commons Attribution License, which permits unrestricted use, distribution, and reproduction in any medium, provided the original work is properly cited.
License URL: https://creativecommons.org/licenses/by/3.0/

Funding: National Institute of Neurological Disorders and Stroke NS02179-02 New York Academy of Medicine

==============================
Miami *Nature Biotechnology* Short Reports
*TheScientificWorld* (2001) 1, 39SR
ISSN 1532-2246

### Bcl-2 SUPPRESSES OXIDATIVE PHOSPHORYLATION DEFECTS CAUSED BY MITOCHONDRIAL DNA MUTATIONS

Giovanni Manfredi[1,]*, Jennifer Q. Kwong[1], Jose A. Oca-Cossio[2], Marilena D'Aurelio[1], Carl D. Gajewsky[1], Flint M. Beal[1], Carlos T. Moraes[2]

[1]Department of Neurology and Neuroscience, Weill Medical College of Cornell University, New York, NY 525 E 68th Street, A-505 New York, NY 10021; [2]Department of Neurology, Miami School of Medicine, University of Miami, Miami, FL
* gim2004@mail.med.cornell.edu

**INTRODUCTION.** The protooncogene Bcl-2 prevents apoptosis by still unknown mechanisms. Bcl-2 has been shown to localize to multiple cell compartments, but mainly to the mitochondria. Bcl-2 has been shown to prevent loss of mitochondrial membrane potential and activation of the mitochondrial permeability transition pore (1), which might be responsible for the activation of some apoptotic pathways. Mutations in the mitochondrial DNA (mtDNA) are associated with a heterogeneous group of sporadic or maternally inherited metabolic disorders. We have analyzed the effects of Bcl-2 overexpression in transmitochondrial cell lines (cybrids) harboring pathogenic mtDNA mutations, to investigate if the Bcl-2 protective effect on mitochondrial function could be extended to primary defects of the mitochondrial respiratory chain.

**METHODS.** Human osteosarcoma 143B $\rho^0$ cells were stably transfected with an eukaryotic expression vector containing the human Bcl-2 cDNA or with vector only. One Bcl-2 expressing clone of 143B $\rho^0$ cells and one containing empty plasmid were selected and repopulated with exogenous mtDNA derived from enucleated cybrids containing either homoplasmic T8356C mtDNA mutation in the $tRNA^{Lys}$ gene (2), or homoplasmic C3256T in the $tRNA^{LeuUUR}$ gene (3), and with the corresponding wild type mtDNA derived from the same patients. ATP synthesis in digitonized whole cells, respiration and respiratory chain activities (4), mitochondrial protein synthesis (5), and complex I and IV subunits steady state levels were analyzed.

**RESULTS.** All Bcl-2 expressing lines showed 10-20 fold higher Bcl-2 levels than mock-transfected cells. Mutated cells overexpressing Bcl-2 showed a 2-8-fold increase in ATP synthesis compared to mock-transfected mutated cells. Oxygen consumption increased to approximately 50% of normal wild type values after Bcl-2 transfection (i.e. 2-5 fold increase). The activities of respiratory chain complexes I+III and IV in mutated cells were partially restored by Bcl-2 overexpression (2-3 fold increase). No increase in respiratory chain activities was observed in wild-type cells transfected with Bcl-2. The activities of the mitochondrial matrix enzymes citrate synthase and succinate dehydrogenase, both encoded by nuclear DNA, were comparable in all cell lines, suggesting that the improvement in enzymatic activities caused by B cl-2 was not due to an increase in the number of mitochondria. The synthesis of mitochondrial polypeptides in T8356C mutated cells was severely reduced, and it was partially increased by Bcl-2 overexpression. Bcl-2

overexpression almost completely restored normal steady state levels of Complex I subunit 1 and complex IV subunits I and II (mtDNA-coded). The levels of complex IV subunit IV and complex I 39-Kda subunit, both encoded by the nuclear DNA, were not increased in cells expressing Bc1-2.

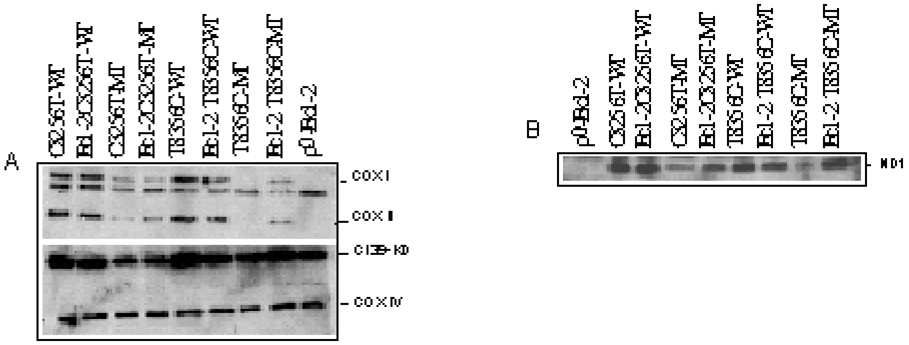

**Steady-state levels of OXPHOS proteins in cell lines overexpressing Bcl-2.** Panel A shows that overexpression of Bcl-2 leads to an increase in steady-state levels of COX I and II in mutant cell lines. Panel B. Because the cell line with a tRNA$^{Leu(UUR)}$ mutation is defective mostly in ND1, a western blot for ND1 was also performed. The figure shows that the steady-state levels of ND1 were also increased by Bcl-2.

**DISCUSSION.** Overexpression of Bcl-2 rescues the defective biochemical phenotype in human cybrid cells harboring pathogenic mutations in mitochondrial tRNA genes. This is a novel function of Bcl-2, which might have important implications for its role in anti-cell death processes and in the cellular response to impaired energy metabolism.

**ACKNOWLEDGEMENTS.** NINDS grant NS02179-02, New York Academy of Medicine Speaker's Fund (G.M.) and CA85700-01 (CTM).

**REFERENCES.**

1. Shimizu, S., Eguchi, Y., Kamiike, W., Waguri, S., Uchiyama, Y., Matsuda, H., and Tsujimoto, Y. (1996) Oncogene 13(1), 21-29
2. Silvestri, G., Moraes, C.T., Shanske, S., Oh, S.J., and DiMauro, S. (1992) Am. J. Hum. Genet. 51(6), 1213-1217
3. Moraes, C.T., Ciacci, F., Bonilla, E., Jansen, C., Hirano, M., Rao, N., Lovelace, R.E., Rowland, L.P., Schon, E.A., and DiMauro, S. (1993) J. Clin. Invest. 92(6), 2906-2915
4. DiMauro, S., Servidei, S., Zeviani, M., DiRocco, M., DeVivo, D.C., DiDonato, S., Uziel, G., Berry, K., Hoganson, G., Johnsen, S.D., et al. (1987) Ann. Neurol. 22(4), 498-506
5. King, M.P., Koga, Y., Davidson, M., and Schon, E.A. (1992) Mol. Cell. Biol. 12(2), 480-490
